# Multiple *Wolbachia* strains provide comparative levels of protection against dengue virus infection in *Aedes aegypti*

**DOI:** 10.1371/journal.ppat.1008433

**Published:** 2020-04-13

**Authors:** Heather A. Flores, Jyotika Taneja de Bruyne, Tanya B. O’Donnell, Vu Tuyet Nhu, Nguyen Thi Giang, Huynh Thi Xuan Trang, Huynh Thi Thuy Van, Vo Thi Long, Le Thi Dui, Huynh Le Anh Huy, Huynh Thi Le Duyen, Nguyen Thi Van Thuy, Nguyen Thanh Phong, Nguyen Van Vinh Chau, Duong Thi Hue Kien, Tran Thuy Vi, Bridget Wills, Scott L. O’Neill, Cameron P. Simmons, Lauren B. Carrington

**Affiliations:** 1 World Mosquito Program, Institute of Vector-Borne Disease, Monash University, Clayton, Victoria, Australia; 2 Oxford University Clinical Research Unit, Hospital for Tropical Disease, Ho Chi Minh City, Vietnam; 3 Hospital for Tropical Diseases, Ho Chi Minh City, Vietnam; 4 Nuffield Department of Medicine, University of Oxford, Oxford, United Kingdom; University of Cambridge, UNITED KINGDOM

## Abstract

The insect bacterium *Wolbachia pipientis* is being introgressed into *Aedes aegypti* populations as an intervention against the transmission of medically important arboviruses. Here we compare *Ae*. *aegypti* mosquitoes infected with *w*MelCS or *w*AlbB to the widely used *w*Mel *Wolbachia* strain on an Australian nuclear genetic background for their susceptibility to infection by dengue virus (DENV) genotypes spanning all four serotypes. All *Wolbachia*-infected mosquitoes were more resistant to intrathoracic DENV challenge than their wildtype counterparts. Blocking of DENV replication was greatest by *w*MelCS. Conversely, *w*AlbB-infected mosquitoes were more susceptible to whole body infection than *w*Mel and *w*MelCS. We extended these findings via mosquito oral feeding experiments, using viremic blood from 36 acute, hospitalised dengue cases in Vietnam, additionally including *w*Mel and wildtype mosquitoes on a Vietnamese nuclear genetic background. As above, *w*AlbB was less effective at blocking DENV replication in the abdomen compared to *w*Mel and *w*MelCS. The transmission potential of all *Wolbachia*-infected mosquito lines (measured by the presence/absence of infectious DENV in mosquito saliva) after 14 days, was significantly reduced compared to their wildtype counterparts, and lowest for *w*MelCS and *w*AlbB. These data support the use of *w*AlbB and *w*MelCS strains for introgression field trials and the biocontrol of DENV transmission. Furthermore, despite observing significant differences in transmission potential between wildtype mosquitoes from Australia and Vietnam, no difference was observed between *w*Mel-infected mosquitoes from each background suggesting that *Wolbachia* may override any underlying variation in DENV transmission potential.

## Author summary

*Aedes aegypti* transmit a number of medically important arboviruses, including dengue, Zika, chikungunya, Mayaro and yellow fever viruses. Over the past 50 years, the burden of *Ae*. *aegypti*-transmitted disease has significantly increased–underscoring how current methods of vector control are unable to cope with this problem. *Wolbachia*-based biocontrol methods show extreme promise in reducing the global burden of vector-borne disease. The *w*Mel strain, widely being used in field trials around the world, substantially reduces the ability of *Ae*. *aegypti* mosquitoes to transmit dengue, Zika, and other viruses. Here we describe a comprehensive comparative study of the viral blocking abilities of *w*Mel to *w*MelCS and *w*AlbB which have previously been shown to have stronger viral blocking or an expanded utility in extreme environments, respectively. Using two different methods to measure viral replication and transmission potential, we show that both strains provide improved viral protection over *w*Mel in the lab supporting further examination in field trials. We further compare the transmission potential of *w*Mel in two different genetic backgrounds and find that *w*Mel provides equivalent levels of viral blocking despite differences observed in wildtype mosquitoes, suggesting that viral blocking induced by *w*Mel may override any underlying variation for DENV transmission potential.

## Introduction

*Aedes aegypti* can transmit a number of medically important arboviruses, including dengue, Zika, chikungunya, Mayaro and yellow fever viruses [1–5]. Together, these viruses cause significant morbidity and mortality across the world, with an estimated 100 million people experiencing a symptomatic dengue virus (DENV) infection each year [6]. Disease control largely relies on methods aimed at suppressing *Ae*. *aegypti* populations. These methods have failed to eliminate dengue as a public health problem in disease endemic countries.

The last decade has seen the field testing of multiple *Wolbachia*-based mosquito biocontrol methods [7,8]. The World Mosquito Program (WMP) has successfully introgressed the *w*Mel strain of *Wolbachia*, originally from *Drosophila melanogaster*, into field populations of *Ae*. *aegypti* [9–12]. *w*Mel has been well-characterised for its capacity to block DENV infection and replication in *Ae*. *aegypti* using different challenge methods [1,13–18]. It demonstrates very effective virus blocking in mosquitoes that have been inoculated with virus, reducing the proportion of mosquitoes that become infected and reducing the tissue viral load (in a variety of tissues) by several orders of magnitude in those that do develop infection [15–17,19]. Further, *w*Mel almost completely reduces the transmission potential of mosquitoes orally challenged with cultured DENV spiked into animal blood [13,15,16,20].

Viremic blood meals from acute dengue patients pose a more rigorous challenge to the virus blocking phenotype mediated by *w*Mel, with some *w*Mel-infected mosquitoes developing infectious saliva [14,15,18]. Nonetheless, this level of *w*Mel-imparted anti-DENV resistance is projected to result in local elimination of DENV transmission in most endemic settings [14].

Other *Wolbachia* strains transinfected into *Ae*. *aegypti* show promise for their enhanced viral blocking abilities or the ability to tolerate extreme environments. The *Wolbachia w*MelCS strain, also from *D*. *melanogaster*, is closely related to *w*Mel [21] and has been successfully transinfected into *Ae*. *aegypti*. *w*MelCS was found to impart similar fitness costs and potentially increased pathogen blocking attributes on *Ae*. *aegypti* as *w*Mel [16]. The *w*AlbB strain, derived from *Ae*. *albopictus* [22], has been shown to block DENV relative to *Wolbachia*-uninfected mosquitoes in lab-based vector competence assays [23]. Comparative studies found *w*AlbB was able to block DENV as effectively as *w*Mel, and characterisation of life history traits showed *w*AlbB resulted in minimal host fitness costs [15,24]. Most notably, *w*AlbB was shown to be more tolerant of cyclical heat stress than *w*Mel, suggesting it may be more stable in areas of high temperature [25,26]. *w*AlbB-infected mosquitoes have recently been released as part of a pilot field trial in Malaysia where the strain was able to successfully establish in the field and passive monitoring suggests a reduction in the incidence of dengue cases [27]. Both strains show promise for their utilisation in *Wolbachia*-based interventions.

In the current study, we perform side-by-side measurements of the susceptibility to DENV infection of *Ae*. *aegypti* lines (Cairns, Australia) infected with *w*Mel, *w*MelCS or *w*AlbB, alongside their uninfected, wildtype counterparts generating one of the most comprehensive and rigorous comparisons to date. First, we perform viral challenges with DENV strains representative of each of the four serotypes circulating in Asia, via intrathoracic inoculation. Second, using data from oral blood feeding with viremic blood from Vietnamese dengue patients, we deliver physiologically relevant insights into the virus transmission potential of each of the three *Wolbachia*-infected strains. We additionally evaluate the effect of host nuclear background on virus susceptibility, comparing *w*Mel and its uninfected control strain on a second genetic background (Ho Chi Minh City, Vietnam) to that from Cairns.

## Results

### Intrathoracic injections with DENV

**Fig 1:**
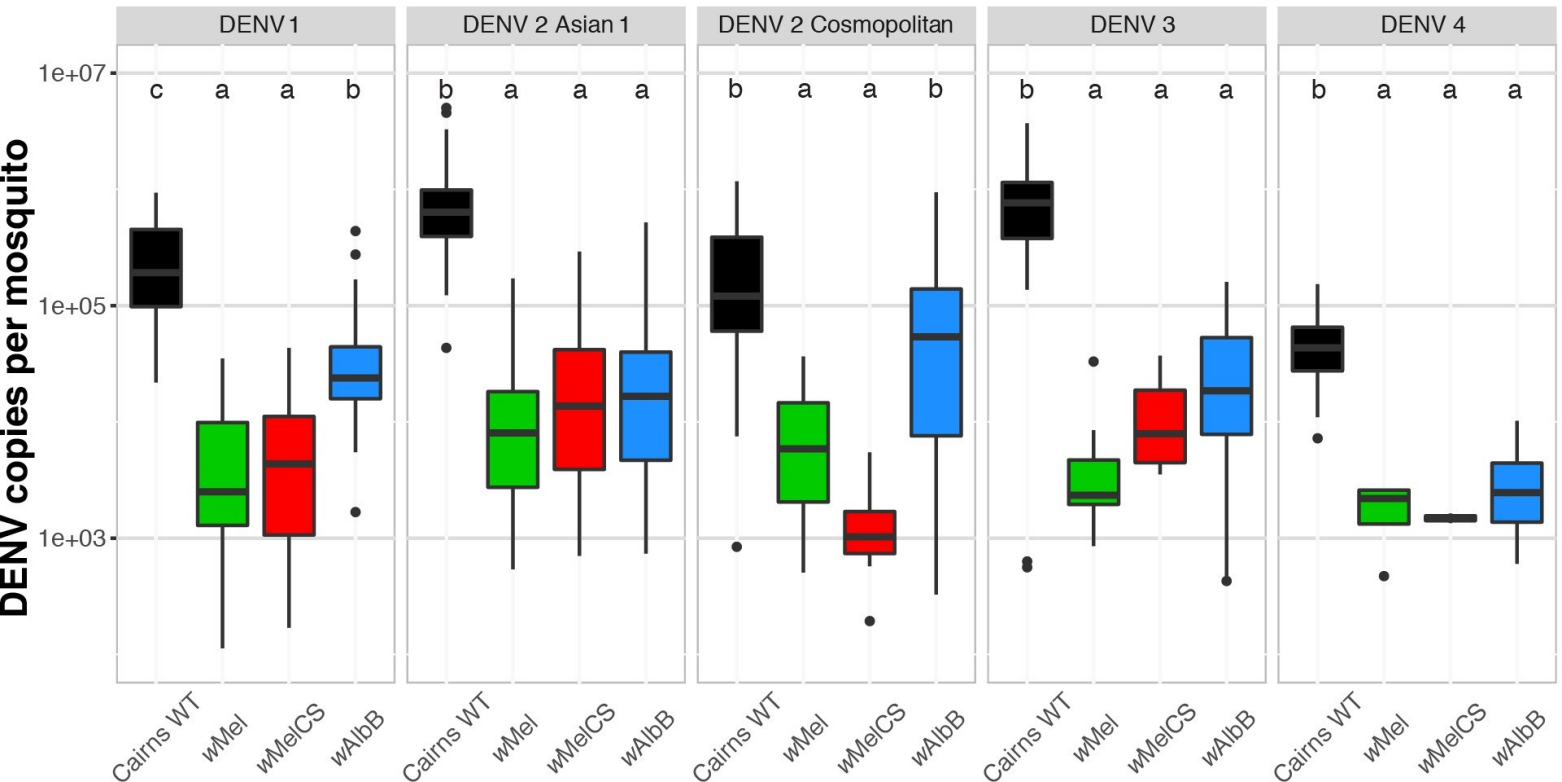
*w*Mel, *w*MelCS, and *w*AlbB all provide viral blocking following intrathoracic DENV infection. DENV-1-4 were intrathoracically injected in 6–7 day-old *w*Mel-, *w*MelCS-, or *w*AlbB-infected mosquitoes as well as wildtype (WT) controls. After a 7-day incubation, RNA was extracted and DENV genomic copies quantified by qRT-PCR. Data are shown as the median DENV genomic copies per mosquito (thick line) ± interquartile ranges (box), extended by the whiskers indicating 1.5× the interquartile range, with dots indicating outliers. *N* for strain-virus combination are shown in Table 1. Using a Kruskal-Wallis test with Dunn’s correction, the letters indicate statistically significant differences between groups.

We generated *Ae*. *aegypti* mosquito lines with *w*Mel, *w*MelCS, or *w*AlbB infections in a genetically similar nuclear background (Cairns, Australia). We compared their susceptibility to DENV infection to that of wildtype (WT) mosquitoes after intrathoracic injection. Mosquitoes were injected with all four DENV serotypes, including two different genotypes of DENV-2, Asian 1 and Cosmopolitan, at concentrations that generally resulted in ~100% infection prevalence amongst WT mosquitoes. We observed that *w*MelCS provided equivalent protection as *w*Mel for all viruses tested. While we did not observe a significant difference in the DENV viral load in *w*MelCS mosquitoes compared to *w*Mel for any virus tested (Kruskal-Wallis test, p > 0.05, **Fig 1**), we observed reductions in the number of mosquitoes acquiring DENV infections (although only for DENV-1 challenge was this statistically significant; see **Table 1,** Fisher’s exact test, p < 0.05). These data suggest *w*MelCS may provide some additional protection over *w*Mel at the level of DENV-1 infection prevalence after intrathoracic injection.

**Table 1:** DENV infection prevalence in *Ae. aegypti* strains with *Wolbachia* infections 6–7 days after intrathoracic injection of DENV-1-4. Significant reductions in the number of infected *w*MelCS mosquitoes relative to *w*Mel is indicated by #, Fisher’s Exact Test. Significant increases in the number of infected *w*AlbB mosquitoes relative to *w*Mel is indicated by ‡, Fisher’s exact test.

	Percentage of mosquitoes PCR-positive for DENV (*N*)			
DENV Strain	Cairns *w*Mel	Cairns *w*MelCS	Cairns *w*AlbB	Cairns WT
DENV-1	94 (48)	51 (47) ^#^	98 (47)	100 (48)
DENV-2 Asian 1	77 (48)	65 (46)	70 (47)	100 (48)
DENV-2 Cosmopolitan	40 (47)	30 (47)	58 (48)	66 (47)
DENV-3	25 (48)	15 (47)	70 (47)^‡^	100 (47)
DENV-4	9 (46)	4 (47)	31 (48)^‡^	100 (47)

*w*AlbB blocked DENV replication relative to Cairns WT, but generally provided less protection than either *w*Mel or *w*MelCS (**Fig 1**). For DENV-1 and DENV-2 (Cosmopolitan genotype) *w*AlbB-infected mosquitoes had a higher DENV viral load than *w*Mel or *w*MelCS-infected mosquitoes (Kruskal-Wallis Test, p < 0.05). Additionally, *w*AlbB mosquitoes had significantly higher infection prevalence compared to *w*Mel when challenged with DENV-3 and DENV-4 (see **Table 1**, Fisher’s exact test, p < 0.05).

### Challenge with acute viremic blood from dengue cases

We used patient-derived blood meals to further discriminate between the effects of *w*Mel, *w*MelCS, *w*AlbB on the Cairns nuclear background (as above), as well as *w*Mel and WT mosquitoes on a Ho Chi Minh City (HCM) nuclear background [18]. The inclusion of these two additional lines enabled us to investigate the influence of mosquito nuclear genetic background on virus susceptibility. All six lines were exposed in parallel to 36 blood meals derived from acute dengue cases, with each of the four DENV serotypes represented. **S2 Table** depicts the flow of human and mosquito samples, data collection and analysis.

**Fig 2:**
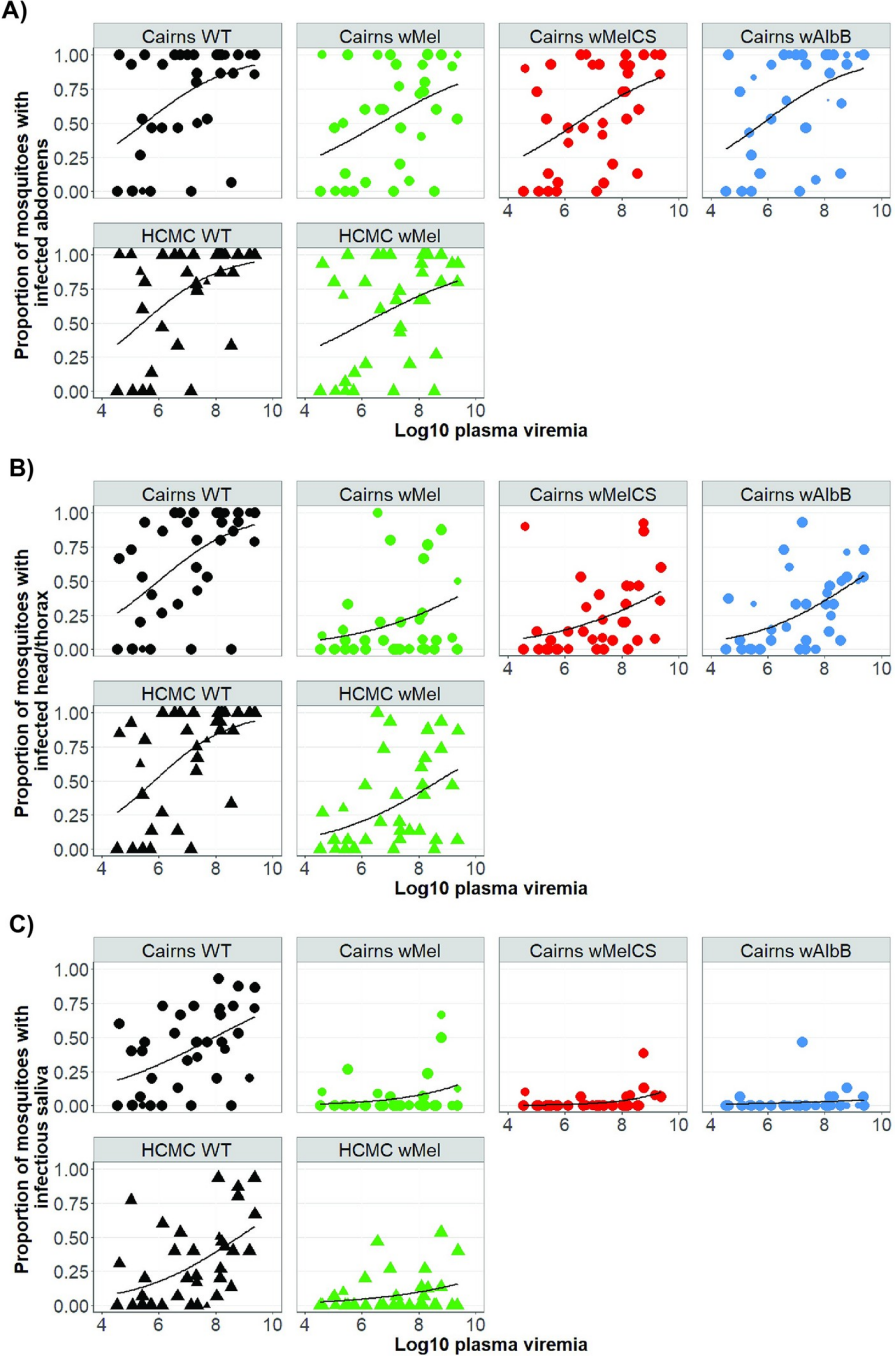
Proportion of mosquitoes from each of the six lines from each cohort with evidence of viral RNA in each tissue tested, after feeding indirectly on patient-derived viremic blood meals. Each dot represents the proportion of each cohort that is infected, plotted as a function of log10 plasma viremia (RNA copies per milliliter). The size of the dot represents the number of mosquitoes tested in each cohort, up to a maximum of 15. Data are stratified by the *Wolbachia* infection status (where “WT” = uninfected, and the host genetic background of the mosquitoes). Figures show percentage of mosquitoes with an infection in A) abdomen tissue; B) head/thorax tissue; and C) naïve mosquitoes that were inoculated with saliva collected from index mosquitoes.

In the 36 patient-derived blood meals, DENV-2 and DENV-4 were most prevalent (n = 14 and 13 respectively), followed by DENV-1 (n = 8) and DENV-3 (n = 1). **S1 Fig** illustrates the range of blood plasma viremias observed in the study. Across the 36 blood feeding events, between 54 and 72% of the mosquitoes from each strain developed an abdomen infection (**Table 2**). Although the magnitude of the effect was not large, all *Wolbachia* strains decreased DENV infection prevalence, compared to Cairns WT mosquitoes (**Fig 2A, S3A Table**)

**Table 2:** Prevalence of DENV infection in the abdomen tissue, head/thorax tissue and saliva-inoculated mosquitoes, measured as a proxy for midgut infection, dissemination and transmission potential, respectively. Mosquitoes had been fed on blood from 36 independent acutely infected dengue patients, and were harvested for collection 14 days after exposure. The number of mosquitoes harvested indicates the total sample size for each strain.

		Percentage of mosquitoes PCR-positive for DENV		
Host background-*Wolbachia* strain	# mosquitoes harvested	Abdomen tissue	Head/thorax tissue	Saliva-inoculated mosquitoes
Cairns WT	523	70.7	65.4	42.3
Cairns *w*Mel	494	54.5	20.0	5.7
Cairns *w*MelCS	521	57.2	22.1	2.7
Cairns *w*AlbB	483	64.4	26.3	2.7
HCM WT	507	71.8	67.7	31.2
HCM *w*Mel	529	60.7	33.3	7.9

In the head/thorax tissues, the prevalence of DENV infection dropped to approximately half of that seen in the abdomen tissue for all *Wolbachia*-infected strains, after 14 days (**Table 2**). There was no difference in the prevalence of DENV infected head/thorax tissues from HCM or Cairns WT mosquitoes (**Fig 2B**).

Finally, we measured the transmission potential of orally-fed mosquitoes by collecting saliva from each harvested female and testing for DENV infection in the pool of recipient mosquitoes into which the saliva sample was inoculated (**Fig 2C**). Overall, DENV transmission potential was lowest for *w*MelCS (14/521 mosquitoes; 2.69%) and *w*AlbB (13/483; 2.69%). The highest prevalence of transmission was observed in the Cairns WT mosquitoes (221/523 = 42.26%) (**Table 2**). In the adjusted marginal logistic regression (**S3C Table**), significant reductions in odds of transmission potential for each of the *Wolbachia* strains relative to WT were observed, with the smallest odds for both Cairns *w*MelCS and *w*AlbB (OR = 0.019, 95% CI = 0.01–0.03, p < 0.001 exactly for both strains). The respective odds for Cairns *w*Mel mosquitoes was only slightly higher (OR = 0.044, 95% CI = 0.03–0.07, p < 0.001).

### Exploratory subgroup analysis

Using the saliva data, we performed subgroup analyses with only the three *Wolbachia* strains introgressed on the Cairns background, to investigate relative strength of the alternate *Wolbachia* strains. Both *w*MelCS (OR = 0.41, 95% CI = 0.20–0.83, p = 0.014) and *w*AlbB (OR = 0.42, 95% CI = 0.20–0.88, p = 0.021) induced greater blocking than *w*Mel, which was used as the reference in this model (**S2 Fig**). We also investigated the effect of host genetic background, and whether *w*Mel’s effectiveness differed between mosquitoes from different origins. In comparing HCM WT mosquitoes with their Cairns counterpart, we found mosquitoes from HCM had lower odds of transmission (OR = 0.499, 95% CI = 0.367–0.681, p < 0.001), with the infection prevalence for Cairns WT mosquitoes 42%, compared to only 31% for HCM WT. Between *w*Mel strains from Cairns and HCM however, there was no observable difference in transmission potential (OR = 1.44, 95% CI = 0.826–2.516, p = 0.198). Among all mosquitoes tested, infectious saliva was detected in 5.6% of mosquitoes with Cairns *w*Mel background and 7.9% for HCM *w*Mel.

### MID_50_ predictions

Using the data from the blood feeding experiments, we predicted the required concentrations of virus in the plasma, measured as RNA copies/mL, to infect 50% of mosquitoes (MID_50_) from each strain, after 14 days (**S3 Fig**). We considered each of the three tissue types in our predictions (abdomen tissue, head/thorax tissue, and saliva). Estimates of the MID_50_ for abdomen tissue of the six strains were fairly similar, ranging from 5.5–6.7 log_10_ viral RNA copies/mL. For detection of virus in saliva, the predicted MID_50_ estimates varied greatly, from 7.9 to 16.4 log_10_ viral RNA copies/mL (**S4 Table**). Because of the wide range of predicted values, with more extreme values associated with each of the *Wolbachia*-infected mosquitoes, we further calculated the MID_10_ and MID_90_, to provide alternative points of comparison. The predicted virus concentrations for the MID_10_ to achieve infectious saliva of *Wolbachia*-infected strains ranged between 8 logs of virus (for HCM *w*Mel) up to 11 logs of virus (for Cairns *w*AlbB). In order to detect virus in the saliva in 90% of each of the *Wolbachia*-infected strains (MID_90_), our model predicts viral concentrations of between 14 logs of virus (Cairns *w*MelCS) and 21.9 logs of virus (for Cairns *w*AlbB) would be needed in the patient-derived blood meal. This is compared to an MID_90_ of <12.5 logs for the WT strains from Cairns and HCM.

### Reduction of viral transmission afforded by *Wolbachia* strains

**Fig 3:**
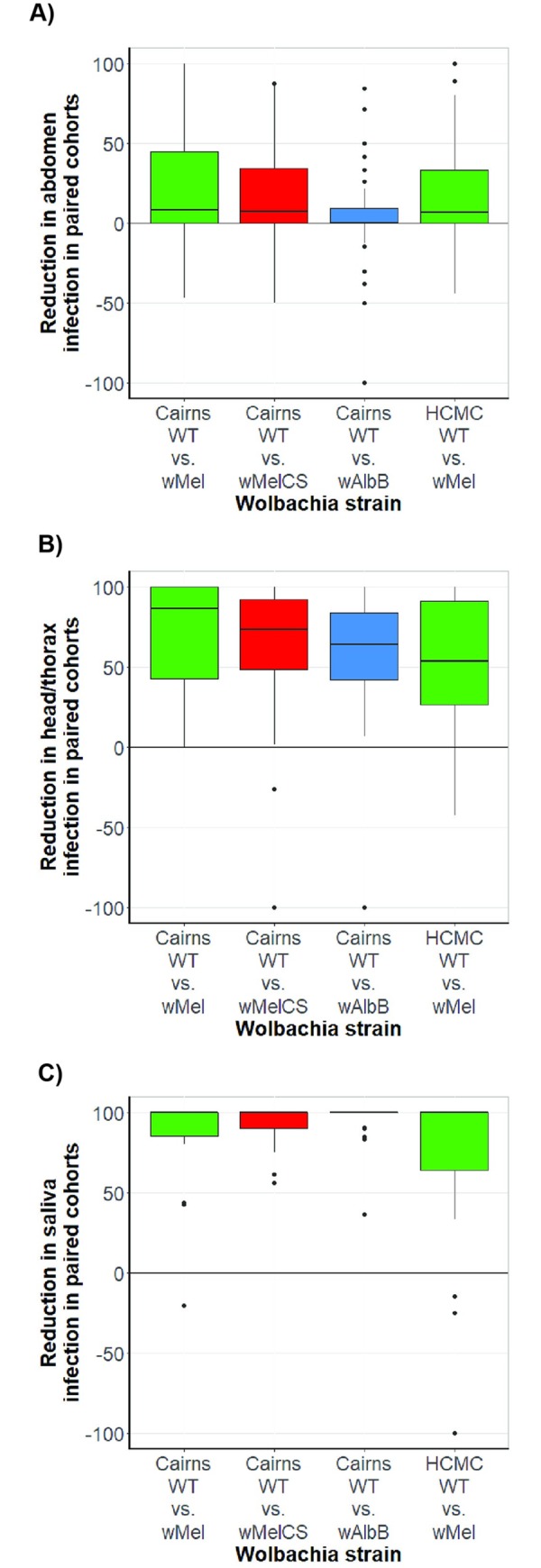
*Wolbachia*-mediated percentage change in infection prevalence of mosquitoes in the *w*Mel, *w*AlbB and *w*MelCS strains on the Cairns and HCM genetic backgrounds. The boxplot depicts the percentage change (medians and interquartile range) in DENV infection prevalence in abdomen, head/thorax, or saliva, between paired WT and *Wolbachia*-infected mosquito cohorts. The y-axis represents the percentage change in infection prevalence, ranging from 100 (meaning all mosquitoes containing *Wolbachia* are DENV-uninfected, and any number of WT mosquitoes are DENV-infected), through 0 (reflecting equal DENV infection prevalence between the paired cohorts), to -100 (reflecting all wildtype mosquitoes being DENV-uninfected, and any number of *Wolbachia*-infected mosquitoes are infected with DENV). Data is stratified by host *Wolbachia* infection and mosquito genetic background. **A)** abdomen infection; **B)** head/thorax infection; **C)** saliva infection, measured by inoculation of saliva into naïve mosquitoes.

We performed pairwise calculations to quantify the magnitude of the effect of each *Wolbachia* strain on virus transmission, between individual cohorts of *Wolbachia*-carrying mosquitoes, relative to their WT controls (**Fig 3**). Differences in DENV infection frequencies in the abdomen tissue between strains were small, but started to emerge between paired cohorts of *Wolbachia*-infected and WT mosquitoes in the head/thorax tissue. Large reductions in transmission potential are induced by all *Wolbachia* strains, but are most prominent in the *w*MelCS and *w*AlbB lines.

## Discussion

Here we provide the first side-by-side comparisons of the pathogen blocking attributes of *w*Mel, *w*MelCS and *w*AlbB in stably transinfected *Ae*. *aegypti*. Relative to the *w*Mel infection in the Cairns background, both *w*MelCS and *w*AlbB provided very small, but measurable, further reductions in the DENV transmission potential of mosquitoes after feeding on viremic blood meals from dengue patients. Subgroup analyses investigating differences between mosquito genetic backgrounds detected reduced virus transmission potential in WT mosquitoes from HCM, compared to those from Cairns. A lack of detectable difference between *w*Mel-infected mosquitoes from these same origins suggests *Wolbachia* may override underlying variation in infection and transmission potential seen between WT populations. Parallel virus injection experiments support the observation that *w*MelCS provides enhanced protection compared to *w*Mel, albeit with a degree of serotype specificity. *w*AlbB in these experiments blocked infection to an intermediate degree only, similar to that seen with abdomen infections in the oral feeding experiments. Mechanistically, this might indicate differences in efficacy between strains and tissue type, and warrants further investigation.

### *Wolbachia* strain performance compared to past studies

While Fraser, *et al*. [16] noted a better relative performance of *w*MelCS over *w*Mel when mosquitoes were challenged with virus by intrathoracic injection, blood feeding studies with blood spiked with cultured virus, noted no difference in the relative performance of *w*Mel and *w*MelCS. Although more costly and difficult to perform, we achieved increased discretionary power using viremic blood meals from dengue patients; at 14 days post-exposure, DENV transmission potential was less in *w*MelCS mosquito cohorts than in *w*Mel cohorts.

Multiple, independent origins of *w*AlbB infections in *Ae*. *aegypti* [17,22] make the comparisons of studies more difficult as *Wolbachia* densities and tropism may underlie the observed differences. Previous work established that *w*AlbB lowers DENV infection and dissemination prevalence relative to uninfected *Ae*. *aegypti* [23]. In a comparison of *Wolbachia* strains, Joubert *et al*. [15] detected similar viral loads in whole mosquito bodies whether they carried *w*Mel or *w*AlbB, after DENV-2 oral challenge (with spiked blood meals). Ant *et al*. [17] found that *w*AlbB had a higher DENV infection prevalence in abdomens than *w*Mel, but lower infection prevalence in salivary glands, although neither was statistically significant. These latter results are consistent with our general findings that *w*AlbB’s inhibition of virus increases as the virus progresses through the mosquito body, surpassing *w*Mel’s levels of viral inhibition. Time-course and anatomical investigations of both DENV and *Wolbachia* are needed to help understand the differences between *w*AlbB’s and *w*Mel’s virus blocking attributes.

*Wolbachia*’s blocking effect is less pronounced when mosquitoes are challenged with DENV serotype 1, but all three *Wolbachia* strains induced particularly effective blocking against DENV-4, in both injection and oral challenge experiments (**Fig 1** and **S2 Fig**). A number of studies [14,18,28,29], have noted that circulating DENV-4 appears to be less infectious to mosquitoes than other serotypes; the consistently high levels of *Wolbachia* blocking against DENV-4 may represent an opportunity to examine mechanisms/pathways involved in DENV interference, driven by virus genotypes and/or *Wolbachia* strains.

### Comparison of inoculation and oral challenge approaches

Injection experiments performed here extend the results of Fraser *et al*. [16], through the addition of *w*AlbB and by employing a more expansive panel of challenge viruses to characterise the viral blocking capacity of *Wolbachia* strains. Our results demonstrate a reasonable correlation between estimates of whole body infection derived from intrathoracic injection of virus and a strain’s relative susceptibility to infection in the abdomen when fed on patient-derived blood. Direct injection of virus into the mosquito thorax bypasses many internal barriers that a virus must clear to be successfully transmitted, likely underestimating a strain’s blocking capacity in the reduction of virus dissemination and transmission. A more appropriate comparison of results might be the contrast between injection results (estimating *Wolbachia*’s ability to block virus prior to dissemination) and the infection prevalence in abdomens after patient-derived blood feeding experiments. Using this approach, we noted that similarly high abdomen infection frequencies for *w*AlbB, relatively to *w*Mel and *w*MelCS, in both injection and oral challenge experiments (see **Tables 1 and 2**).

### Considerations for *Wolbachia* deployment

Multiple studies suggest that *w*Mel, *w*MelCS, and *w*AlbB have mild impacts on *Ae*. *aegypti* fitness [1,15,17,22,24]. Given the successful introgression of *w*Mel and *w*AlbB into field populations thus far [9–12,27], we expect all three strains to perform well in the field. A major concern for *Wolbachia*-based interventions is the attenuation of virus blocking. Two main considerations here are the likelihood of attenuation affecting all *Wolbachia* strains and how alternative *Wolbachia* strains can be deployed to replace less effective ones.

Studies thus far have not found a single mechanism of major effect that underlies *Wolbachia*-mediated viral blocking. Multiple mechanisms, including competition over resources and immune priming, are suggested to contribute to blocking [30–32]. As such, attenuation of the blocking phenotype for all *Wolbachia* strains is unlikely. However, if a single mechanism of major effect were employed by *Wolbachia* strains across supergroups to interfere with virus replication and transmission, and DENV evolves to evade this mechanism, then the capacity to use replacement strains as part of a resistance management strategy is diminished. With deployment of suitable replacement strains, any extension of the strategy’s longevity allows additional time for improving and evaluating other vaccine candidates, and development of alternative vector control strategies. Efficient replacement of an existing *Wolbachia* strain requires the cytoplasmic incompatibility (CI) loci of the replacement strain to be uni- or bidirectionally incompatible with those of the primary strain. The observed bi-directional CI phenotype of *w*AlbB with both *w*Mel and *w*MelCS suggests a number of options for secondary releases [15,16] should they be needed to overcome any attenuation of the desired viral blocking phenotype. Nevertheless, investigations into the mechanisms by which each strain inhibits virus infection, replication and transmission should thus be a heightened priority to help gauge the long-term stability of *Wolbachia*’s antiviral effect in this context.

In summary, our data support the use of *w*MelCS and *w*AlbB in first-line releases to reduce dengue burden in endemic countries, or as part of management strategies in the event of any attenuation of the virus-blocking phenotype. Given the effective performance of all strains, and that lab-based studies suggest *w*AlbB may have a broader optimal temperature range [25,26] there is a suite of strains feasibly available for deployment in an expanded range of environmental conditions. While our data suggest that *w*MelCS and *w*AlbB provide a small reduction in DENV transmission potential compared to *w*Mel, the operational impact of this difference is not clear. Field investigations to monitor the establishment and stability of each strain across variable environmental conditions as well as the impact on dengue incidence are warranted to understand if these differences show any significance in the field.

## Materials and methods

There were two parallel experiments conducted in this study. The injection experiments were conducted at Monash University, Melbourne, Australia, while the vector competence experiments were performed at Oxford University Clinical Research Unit (OUCRU), in Ho Chi Minh City, Vietnam.

### Ethics statement

Blood feeding of mosquito colonies at Monash University on adult, human volunteers was performed in accordance with Monash University Human Research Ethics permit number CF11/0766-2011000387. Written informed consent was provided by all volunteers prior to commencement. Although ethics approval was not required for blood feeding of mosquito colonies in Vietnam, adult volunteers were still requested to sign an informed consent form prior to their participation. All patients participating in the clinical observation study in Vietnam were prospectively enrolled as part of a study approved by the ethics committees of the Hospital for Tropical Diseases (CS/NÐ/16/27), Oxford Tropical Research Ethics Committee (45–16), and University of Melbourne Human Research Ethics Committee (1648095). These approvals allowed patients admitted to the Hospital for Tropical Diseases, who were >15 years of age, had been unwell for <96 hrs, and were suspected to have dengue to provide a single venous blood sample for use in mosquito feeding. Written informed consent was prospectively obtained from all participants by qualified staff from the hospital.

### Generation of mosquito lines

#### Cairns background mosquitoes

The *w*Mel and *w*MelCS strains have been previously described [1,16]. To generate the *w*AlbB strain in an Australian *Ae*. *aegypti* genetic background, *w*AlbB from the WB1 strain [22] was injected into *w*MelF.Tet, an uninfected, genetically Australian strain. Embryonic micro-injections, creating and establishing isofemale lines were performed as previously described [16].

A wildtype (WT) colony was established by placing ovitraps in Bentley Park and Edmonton (Cairns, Australia) in September 2016. Eggs were hatched in the lab and reared to adult stage where they were sorted by species and used to establish a single colony. Eggs from the WT colony were used in experiments within three generations of colony establishment. *w*Mel, *w*MelCS, and *w*AlbB strains were backcrossed to the Cairns WT colony for three generations to isogenize the genetic backgrounds of the three different strains.

For colony maintenance, all mosquitoes were reared as described previously, with minor differences [33]. Briefly, adult mosquitoes were maintained in a controlled temperature room at 26°C with 65% relative humidity (RH) with 12h:12h light-dark cycle and were allowed access to 10% sucrose *ad libitum*, as well as to human blood for reproduction.

Subcultures of these colonies were delivered to OUCRU for the blood feeding experiments. At OUCRU, colony maintenance was performed at ~28°C, 75–85% RH and 12h:12h light-dark cycle. Genetic material was obtained approximately every 3 months from the Monash colonies. *Wolbachia* infection status was confirmed before using the new material, as well as in each subsequent generation of maintenance, to ascertain there was no cross-contamination of *Wolbachia* strains between cages, or failure of maternal transmission between generations.

#### Ho Chi Minh City (HCM) background mosquitoes

The Ho Chi Minh City (HCM) WT mosquitoes were freshly collected from the field and then colonised in the laboratory as described in [34]. The HCM *w*Mel line was originally produced with backcrossing of the *Wolbachia* infected Cairns females with HCM WT males, for five generations. From this point both the HCM WT and HCM *w*Mel lines underwent outcrossing with 10–20% field-derived (F_1_) males every second generation in order to maintain genetic similarity with the local field population. As per the Cairns mosquitoes, both lines had their *Wolbachia* infection status confirmed by PCR each generation. The HCM WT and *w*Mel lines were maintained in duplicate cages, of >250 females/cage, at ~28°C, 75–85% RH and a 12:12 hr light dark cycle. Mosquitoes used in blood feeding experiments were from generations 28–34 for HCM WT and 33–39 for HCM *w*Mel.

#### Intrathoracic injections

Asian isolates of DENV 1–4 were obtained from the World Reference Center for Emerging Viruses and Arboviruses (WRCEVA) and from the Oxford University Clinical Research Unit, Vietnam. Virus genotype and origin are listed in **S1 Table**. Virus genotypes were confirmed by PCR and sequencing of the E gene. C6/36 cells were infected at a MOI of 0.1, and the cell culture supernatant was harvested 7 days later. Virus concentrations were determined by TCID_50_ using monoclonal antibody 4G2 (provided by Roy Hall), followed by incubation with HRP-conjugated secondary antibodies, and TMB substrate. Mosquitoes used for injection experiments were from generations G2-G5 after the completion of backcrossing for all *Wolbachia* infected strains, and G4-G7 for Cairns WT.

Mosquitoes were age controlled within 24 hours of one another to minimize experimental variation. For viral injections, 6–7 day old mosquitoes were intrathoracically injected with 69 nL of virus diluted in RPMI to the concentrations listed in **S1 Table** using a microinjector (Nanoject III, Drummond Scientific) with pulled-glass capillary needles. Mosquitoes were then incubated as per their standard rearing conditions for 7 days before collecting whole mosquitoes and testing them individually for infection status.

To quantify viral genomic copies, total RNA was extracted from mosquitoes using RNeasy 96 QIAcube HT kits (QIAGEN). DENV genome copies were quantified using pan-DENV primers that bind the DENV 3’UTR [16,35] and LightCycler Multiplex RNA Virus Master (Roche) one-step qRT-PCR mix.

### Blood feeding experiments

#### Viremic blood meals for mosquitoes

A total of 42 venous blood meals from acute dengue cases were provided to the mosquitoes. An aliquot of the blood draw from each patient was used to determine the DENV serotype and viremia level, using a serotype-specific PCR (see Diagnostics section below). The viremia level in six blood meals was below the limit of PCR detection and were excluded from the analysis. Amongst these six feeds, we detected DENV PCR-positive mosquitoes from three of the blood meals. Applying our serotype-specific PCR on respective mosquito samples, we confirmed all three patients were infected with DENV-2. However, due to our inability to measure viremia of the original blood meal, a factor shown to increase the likelihood of subsequent virus transmission by the mosquito [34], we excluded from the analysis all six blood meals and associated mosquitoes (**S2 Table**).

#### Mosquito infection experiments

Mosquitoes 1–4 days old were exposed to dengue viremic blood using an artificial membrane feeder, for 30 mins. Up to 30 engorged mosquitoes were collected and retained for incubation, of which a maximum of 15 surviving mosquitoes were selected randomly for collection after 14 days. Abdomen, head/thorax and saliva samples were harvested, and processed as per Carrington *et al*. [18]. Saliva samples were inoculated into naïve WT HCM mosquitoes to confirm the presence of infectious DENV particles. Inoculated WT mosquitoes were harvested 7 days post-injection and processed [18].

#### PCR diagnostics

Mosquitoes were homogenized with a single bead in squash buffer (containing Tris base, EDTA, NaCl, and proteinase K), heated at 56°C for 10 min and 98°C for 15 min, before being cooled to 15°C. Samples were centrifuged for 2 min to pellet debris and 2 μL of the clarified sample was added to the PCR. PCR confirmation of mosquito tissue samples for DENV and *Wolbachia* infection status was performed using the combined amplification results of two PCRs for each sample. The first (duplex) one-step qRT-PCR targeted DENV using pan-serotype primers [16,35], and an *Ae*. *aegypti* internal control, *RPS17*. The second (multiplex) qPCR was used to confirm the infecting *Wolbachia* strain. This *Wolbachia* PCR targeted *w*Mel, *w*MelPop (which also amplifies *w*MelCS), and *w*AlbB, as well as the *Ae*. *aegypti* internal control; the specific combination of positive results was used to determine the infection status. Across both PCRs, the internal control had to be positive in order to accept the results as valid.

Mosquito samples were tested by technicians blinded to the serotype and viremia information of the associated plasma sample. The samples were tested sequentially (abdomen, head/thorax and then the pooled inoculated mosquitoes). If abdomen tissue was DENV-positive, both the head/thorax and saliva samples were subsequently processed. If the abdomen was DENV-negative, then the following tissues were assumed to be uninfected, and not tested. Mosquito samples were re-tested if the PCR results were not congruent (eg: negative for DENV in the head/thorax, but positive in saliva). Samples were eventually excluded from the analysis if re-testing remained incongruent.

Mosquitoes were determined to have: a *w*Mel infection if they were *w*Mel(+), *w*MelPop(-), and *w*AlbB(-); a *w*MelCS infection if they were *w*Mel(+), *w*MelPop(+), and *w*AlbB(-); or a *w*AlbB infection if they were *w*Mel(-), *w*MelPop(-) and *w*AlbB(+). WT samples were negative for all *Wolbachia* targets. Any samples with combinations of results other than those listed above, or where the *Wolbachia* infection results between the abdomen and thorax samples did not match, were excluded from the analysis.

All primer sets have been previously described [36], except those targeting *w*AlbB, which were: Forward “Alb_16009_F” primer (5'-AGTAGTGCAGCGAGTCT-3'), Reverse “Alb_16009_R” primer (5'-AGTTCACTGTGCTACTTGCCA-3'), and Probe “Alb_16009_LNA500” (5'-Cyan500-TATCCCCT+ACC+A+A+AGC+AAT-BHQ1-3’). PCR-positivity for viral RNA was based on a Ct value of 35 or less for all targets.

Viral RNA was extracted from human plasma samples using MagNA 96 extraction kits (Roche). Virus sample serotyped using a validated, quantitative serotype-specific RT-PCR [37], with viremia calculated based on ratios between genome copies per mL and plaque forming units per mL, of 214:1 for DENV-1, 73:1 for DENV-2, 436:1 for DENV-3, and 101:1 for DENV-4 [37]. A subset of mosquito samples were also tested using this PCR method, when it was not possible to determine the serotype of the infecting virus using the plasma sample directly but it was observed that the mosquitoes still became infected.

### Statistical analyses

For intrathoracic injection experiments, viral genomic copies per mosquito were plotted as medians (± interquartile ranges) using boxplots excluding mosquitoes negative for virus. Significant differences in viral copy numbers were determined using Kruskal-Wallis tests with Dunn’s multiple comparison correction. Significant differences in DENV infection prevalence relative to *w*Mel were calculated using one-tailed Fisher’s exact tests.

The risk of human-to-mosquito infection was assessed using marginal logistic regression models. An unadjusted, baseline model accounting for *Wolbachia* strain was considered, as well as an adjusted model, accounting for *Wolbachia* strain, infecting virus serotype, and viremia. Infection frequencies for each cohort of mosquitoes were plotted as a function of plasma viremia, and logistic curves overlaid. For each blood meal we calculated the relative reductions in the proportion of mosquitoes that developed an infection, between each *Wolbachia* strain and its WT counterpart of the same host background, and plotted the medians (±IQRs) of these differences using boxplots. Finally, using marginal logistic regression modelling, we calculated the infectious dose required for 50% of the mosquitoes (Mosquito infectious dose, MID_50_) from each strain to a) become infected with virus, b) disseminate the virus in head/thorax tissue and c) have evidence of virus in saliva. Given the low prevalence of virus infection in strains containing *Wolbachia* in the head/thorax tissue and saliva, estimates of the MID_50_ often went beyond the range of viremias observed in the study, thus giving unacceptably large confidence intervals. Thus, we provide additional estimates of the more extreme MIDs, to achieve 10% and 90% infection as well (**S4 Table**).

## Supporting information

S1 Table List of virus isolates, their origins and infecting dose, used in the injection experiments against four Cairns mosquito lines (Cairns WT, Cairns *w*Mel, Cairns *w*MelCS, Cairns *w*AlbB). WRCEVA = World Reference Center for Emerging Viruses and Arboviruses. (DOCX)

S2 Table Table describing the exclusion of data from the analysis in the direct feeding experiment, and the associated reasons for the exclusion. LOD = limit of PCR detection.
(DOCX)

S3 Table Adjusted marginal logistic regression models for the risk of viral infection in the abdomen tissue (A), head/thorax tissue (B); and mosquitoes inoculated with saliva (C). The reference categories for each covariate are listed in the tables. *NB*: There was only a single patient blood meal containing DENV-3, therefore the confidence intervals surrounding the Odds Ratio is extremely large.
(DOCX)

S4 Table Calculated concentration of circulating virus in the patient blood (measured as log_10_ RNA copies/mL in patient plasma) required to infect the abdomen, head/thorax and saliva of mosquitoes from each of the six mosquito strains assessed in this study. The viremias required to achieve 10%, 50% and 90% of mosquitoes (the MID_10_, MID_50_ and MID_90_ respectively) with evidence of virus in each tissue type are calculated for each line.
(DOCX)

S1 Fig Boxplots showing the median ± IQR of viremias from patient derived blood meal. Viremia was measured by qRT-PCR, and reported as log10 RNA copies/mL for the 36 blood meals to which a serotype and viremia could be measured.
(TIF)

S2 Fig Proportion of mosquitoes from each of the three Cairns strains carrying *Wolbachia*, with infectious virus in their saliva, after feeding on patient-derived viremic blood meals. Each dot represented the proportion of each cohort that is infected, plotted as a function of log_10_ plasma viremia (RNA copies per milliliter), with the size of the dot indicative of the number of mosquitoes tested in each cohort, up to a maximum of 15. Data are stratified by the *Wolbachia* infection status, and the infecting serotype in the patient blood meal.
(TIF)

S3 Fig Predicted concentrations of virus leading to mosquitoes with DENV-positive (A) abdomens (representing midgut infections), (B) head/thorax (disseminated infections), and (C) infectious saliva (as measured in saliva-inoculated mosquitoes). Each point represents the predicted proportion of all mosquitoes to have DENV in the respective tissue tested, 14 days after a blood meal on a viremic blood from a dengue patient. The corresponding smoothing curves and shading (representing 95% CIs) illustrate the predicted probability based on marginal logistic regression. The point at which the smoothing curves cross the 50% on the y-axis represents the predicted concentration of virus required to infect 50% of mosquitoes (50% Mosquito Infection Dose; MID_50_).
(TIF)

S1 Data All raw data are available within S1 Data. (XLSX)
