# Supplementary material for: Multiple Wolbachia strains provide comparative levels of protection against dengue virus infection in Aedes aegypti
Source: PLoS Pathog. 2020 Apr 13;16(4):e1008433. doi: 10.1371/journal.ppat.1008433 (PMC7179939; doi:10.1371/journal.ppat.1008433)
Supplement: S1 Table — (DOCX) [file ppat.1008433.s001.docx]

S1 Table: List of virus isolates, their origins and infecting dose, used in the injection experiments against four Cairns mosquito lines (Cairns WT, Cairns *w*Mel, Cairns *w*MelCS, Cairns *w*AlbB). WRCEVA = World Reference Center for Emerging Viruses and Arboviruses.

| **Strain** | **Genotype** | **Isolated** | **Titre used for injections** | **Genbank Acc. #** | **Obtained from** |
| --- | --- | --- | --- | --- | --- |
| DENV-1 | Genotype 1 | Vietnam 2008 | 1.2 x 10^5^ TCID_50_/ml | FJ461335 | WRCEVA |
| DENV-2 | Asian 1 | Vietnam 2010 | 2.4 x 10^5^ TCID_50_/ml | NA | Isolated in Vietnam, 2010 |
| DENV-2 | Cosmopolitan | Vietnam 2006 | 3.4 x 10^5^ TCID_50_/ml | EU482672 | WRCEVA |
| DENV-3 | Genotype 2 | Myanmar 2008 | 4.5 x 10^3^ TCID_50_/ml | KT452792 | WRCEVA |
| DENV-4 | Genotype 1 | Cambodia 2011 | 8.0 x 10^5^ TCID_50_/ml | KT452802 | WRCEVA |
