# Supplementary material for: Multiple Wolbachia strains provide comparative levels of protection against dengue virus infection in Aedes aegypti
Source: PLoS Pathog. 2020 Apr 13;16(4):e1008433. doi: 10.1371/journal.ppat.1008433 (PMC7179939; doi:10.1371/journal.ppat.1008433)
Supplement: S2 Table — LOD = limit of PCR detection. (DOCX) [file ppat.1008433.s002.docx]

S2 Table: Table describing the exclusion of data from the analysis in the direct feeding experiment, and the associated reasons for the exclusion. LOD = limit of PCR detection.

|  | **Patients** | | **Mosquitoes** | |
| --- | --- | --- | --- | --- |
| **Starting number** |  | **42** |  | **3600** |
|  | **Excluded** | **Remaining** | **Excluded** | **Remaining** |
| Excluding patients with unconfirmed serotype | 3 | 39 | 266 | 3334 |
| Excluding patients with viremia of below LOD* | 3 | 36 | 257 | 3077 |
| Mosquitoes with irregular *Wolbachia* status |  |  | 1 | 3076 |
| Mosquitoes with irregular DENV infection status |  |  | 11 | 3065 |
| Mosquitoes found dead at day of harvest |  |  | 7 | 3058 |
| Mosquitoes unaccounted for (lost) |  |  | 1 | 3057 |
| **Final data set for analysis** | 6 | **36** | 543 | **3057** |
| *Plasma samples were negative by serotype-specific PCR. Serotype was confirmed for these samples by determining the serotype of infected mosquitoes | | | | |
