# Supplementary material for: Multiple Wolbachia strains provide comparative levels of protection against dengue virus infection in Aedes aegypti
Source: PLoS Pathog. 2020 Apr 13;16(4):e1008433. doi: 10.1371/journal.ppat.1008433 (PMC7179939; doi:10.1371/journal.ppat.1008433)
Supplement: S3 Table — Adjusted marginal logistic regression models for the risk of viral infection in the abdomen tissue (A), head/thorax tissue (B); and mosquitoes inoculated with saliva (C). The reference categories for each covariate are listed in the tables. NB: There was only a single patient blood meal containing DENV-3, therefore the confidence intervals surrounding the Odds Ratio is extremely large. (DOCX) [file ppat.1008433.s003.docx]

S3 Table: Adjusted marginal logistic regression models for the risk of viral infection in the abdomen tissue (A), head/thorax tissue (B); and mosquitoes inoculated with saliva (C). The reference categories for each covariate are listed in the tables. *NB*: There was only a single patient blood meal containing DENV-3, therefore the confidence intervals surrounding the Odds Ratio is extremely large.

| 1. **Abdomen tissue** | **Odds ratio** | **95% conf. int.** | ***p* value** |
| --- | --- | --- | --- |
| (Intercept) | 4.1E-04 | (4.7E-06; 0.04) | **0.001** |
| Cairns WT (reference) |  |  |  |
| Cairns *w*Mel | 0.22 | (0.15; 0.33) | **<0.001** |
| Cairns *w*MelCS | 0.29 | (0.2; 0.43) | **<0.001** |
| Cairns *w*AlbB | 0.67 | (0.45; 1) | **0.048** |
| HCM WT | 1.16 | (0.76; 1.77) | 0.499 |
| HCM *w*Mel | 0.34 | (0.23; 0.5) | **<0.001** |
| DENV-1 (reference) |  |  |  |
| DENV-2 | 1.26 | (0.16; 9.7) | 0.826 |
| DENV-3 | 5.0E+07 | (0.00; ∞) | 0.987 |
| DENV-4 | 0.06 | (0.01; 0.5) | **0.009** |
| Log_10_ plasma viremia (+1) | 4.16 | (2.33; 7.44) | **<0.001** |

| 1. **Head/thorax tissue** | **Odds ratio** | **95% conf. int.** | ***p* value** |
| --- | --- | --- | --- |
| (Intercept) | 8.1E-04 | (0; 0.02) | **<0.001** |
| Cairns WT (reference) |  |  |  |
| Cairns *w*Mel | 0.04 | (0.03; 0.06) | **<0.001** |
| Cairns *w*MelCS | 0.04 | (0.03; 0.06) | **<0.001** |
| Cairns *w*AlbB | 0.06 | (0.04; 0.09) | **<0.001** |
| HCM WT | 1.25 | (0.86; 1.8) | 0.240 |
| HCM *w*Mel | 0.09 | (0.07; 0.13) | **<0.001** |
| DENV-1 (reference) |  |  |  |
| DENV-2 | 0.93 | (0.21; 4.06) | 0.922 |
| DENV-3 | 32.29 | (1; 1044.79) | 0.050 |
| DENV-4 | 0.11 | (0.02; 0.49) | **0.004** |
| Log_10_ plasma viremia (+1) | 3.40 | (2.21; 5.22) | **<0.001** |

| 1. **Mosquitoes inoculated with saliva** | **Odds ratio** | **95% conf. int.** | ***p* value** |
| --- | --- | --- | --- |
| (Intercept) | 3.8E-03 | (0; 0.06) | **<0.001** |
| Cairns WT (reference) |  |  |  |
| Cairns *w*Mel | 0.04 | (0.03; 0.07) | **<0.001** |
| Cairns *w*MelCS | 0.02 | (0.01; 0.03) | **<0.001** |
| Cairns *w*AlbB | 0.02 | (0.01; 0.03) | **<0.001** |
| HCM WT | 0.50 | (0.37; 0.68) | **<0.001** |
| HCM *w*Mel | 0.06 | (0.04; 0.09) | **<0.001** |
| DENV-1 (reference) |  |  |  |
| DENV-2 | 0.76 | (0.23; 2.55) | 0.655 |
| DENV-3 | 3.99 | (0.24; 65.11) | 0.331 |
| DENV-4 | 0.31 | (0.09; 1.05) | 0.061 |
| Log_10_ plasma viremia (+1) | 2.17 | (1.52; 3.09) | **<0.001** |
