# Supplementary material for: Multiple Wolbachia strains provide comparative levels of protection against dengue virus infection in Aedes aegypti
Source: PLoS Pathog. 2020 Apr 13;16(4):e1008433. doi: 10.1371/journal.ppat.1008433 (PMC7179939; doi:10.1371/journal.ppat.1008433)
Supplement: S4 Table — The viremias required to achieve 10%, 50% and 90% of mosquitoes (the MID10, MID50 and MID90 respectively) with evidence of virus in each tissue type are calculated for each line. (DOCX) [file ppat.1008433.s004.docx]

S4 Table: Calculated concentration of circulating virus in the patient blood (measured as log_10_ RNA copies/mL in patient plasma) required to infect the abdomen, head/thorax and saliva of mosquitoes from each of the six mosquito strains assessed in this study. The viremias required to achieve 10%, 50% and 90% of mosquitoes (the MID10, MID50 and MID90 respectively) with evidence of virus in each tissue type are calculated for each line.

|  |  |  | **Infected abdomen tissue** | | | **Infected head/thorax tissue** | | | **Infection in saliva** | | |
| --- | --- | --- | --- | --- | --- | --- | --- | --- | --- | --- | --- |
|  | **Strain** |  | **MID_10_** | **MID_50_** | **MID_90_** | **MID_10_** | **MID_50_** | **MID_90_** | **MID_10_** | **MID_50_** | **MID_90_** |
| **Cairns** | **WT** | Estimate | 1.99 | 5.50 | 9.02 | 2.23 | 5.91 | 9.58 | 3.41 | 7.89 | 12.36 |
|  |  | (95% CI) | (-2.00;5.98) | (4.14;6.87) | (6.80;11.23) | (-1.51;5.97) | (4.81;7.00) | (7.10;12.07) | (1.10;5.72) | (7.06;8.71) | (9.35;15.37) |
|  | ***w*Mel** | Estimate | 1.87 | 6.70 | 11.54 | 5.07 | 11.23 | 17.38 | 8.57 | 12.12 | 15.68 |
|  |  | (95% CI) | (-3.58;7.31) | (5.57;7.83) | (6.98;16.09) | (1.43;8.70) | (4.60;17.85) | (1.24;33.53) | (6.97;10.18) | (6.43;17.82) | (5.49;25.86) |
|  | ***w*AlbB** | Estimate | 2.19 | 5.81 | 9.42 | 4.84 | 9.43 | 14.01 | 11.01 | 16.44 | 21.86 |
|  |  | (95% CI) | (-1.35;5.73) | (4.63;6.98) | (7.1;11.74) | (2.65;7.03) | (7.64;11.22) | (8.90;19.13) | (6.08;15.95) | (2.98;29.90) | (-0.49;44.22) |
|  | ***w*MelCS** | Estimate | 2.25 | 6.47 | 10.68 | 5.14 | 10.43 | 15.72 | 9.31 | 11.65 | 13.99 |
|  |  | (95% CI) | (-1.87;6.38) | (5.37;7.56) | (7.53;13.82) | (1.90;8.38) | (6.15;14.71) | (4.44;26.99) | (8;10.62) | (8.45;14.85) | (8.76;19.22) |
| **HCM** | **WT** | Estimate | 2.35 | 5.53 | 8.71 | 2.73 | 5.87 | 9.01 | 5.10 | 8.69 | 12.27 |
|  |  | (95% CI) | (-1.00;5.69) | (4.27;6.79) | (6.98;10.45) | (-0.33;5.79) | (4.8;6.94) | (7.28;10.75) | (3.08;7.13) | (7.78;9.59) | (9.09;15.45) |
|  | ***w*Mel** | Estimate | 0.92 | 6.17 | 11.42 | 4.06 | 8.99 | 13.92 | 8.12 | 12.49 | 16.86 |
|  |  | (95% CI) | (-5.73;7.58) | (4.64;7.70) | (6.67;16.16) | (0.78;7.35) | (6.76;11.22) | (6.81;21.03) | (6.80;9.43) | (7.00;17.98) | (6.33;27.4) |
