# Supplementary figures and images for: Multiple Wolbachia strains provide comparative levels of protection against dengue virus infection in Aedes aegypti
Source: PLoS Pathog. 2020 Apr 13;16(4):e1008433. doi: 10.1371/journal.ppat.1008433 (PMC7179939; doi:10.1371/journal.ppat.1008433)

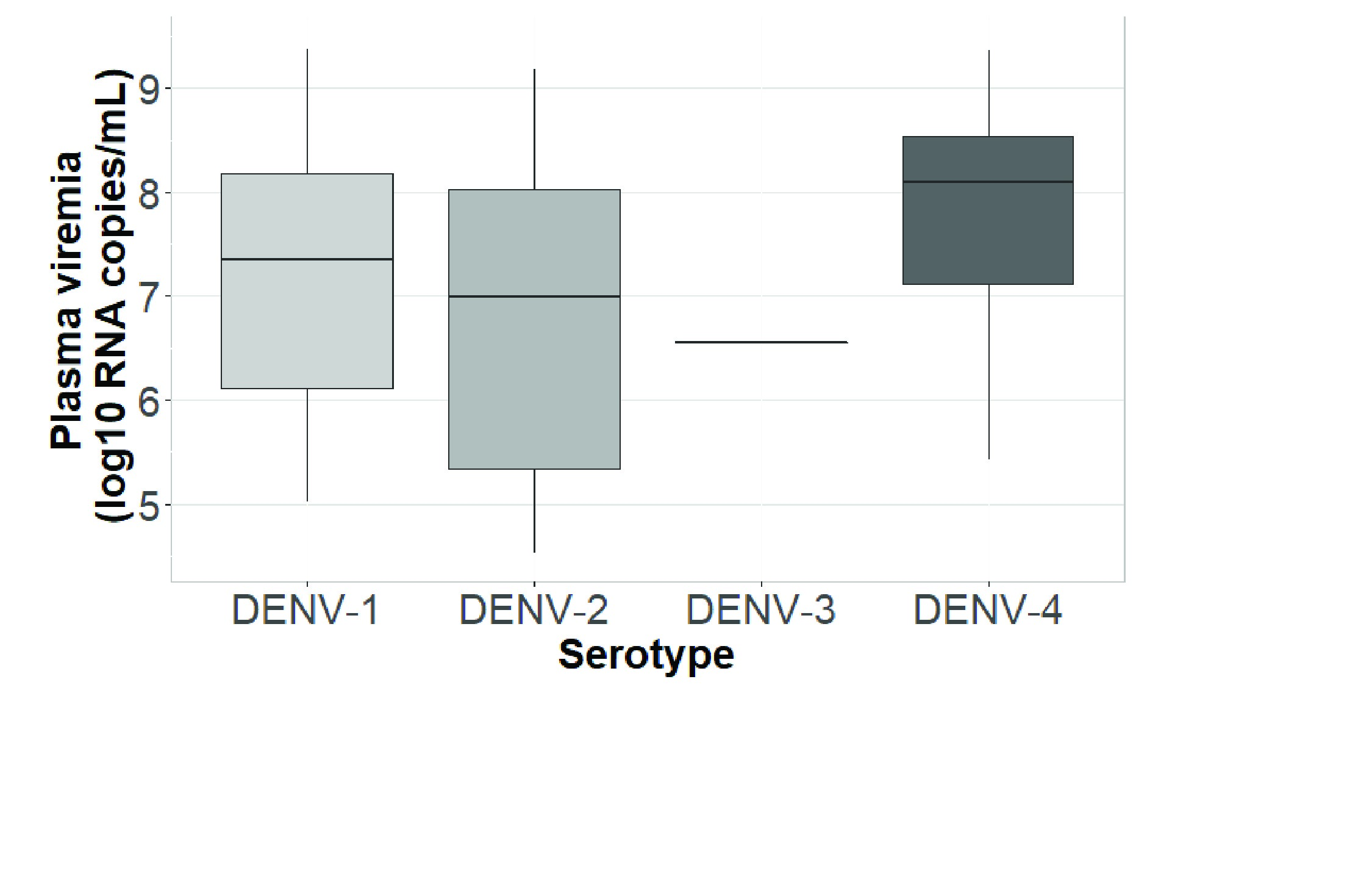

Supplement: S1 Fig — Viremia was measured by qRT-PCR, and reported as log10 RNA copies/mL for the 36 blood meals to which a serotype and viremia could be measured. (TIF) [file ppat.1008433.s005.tif]

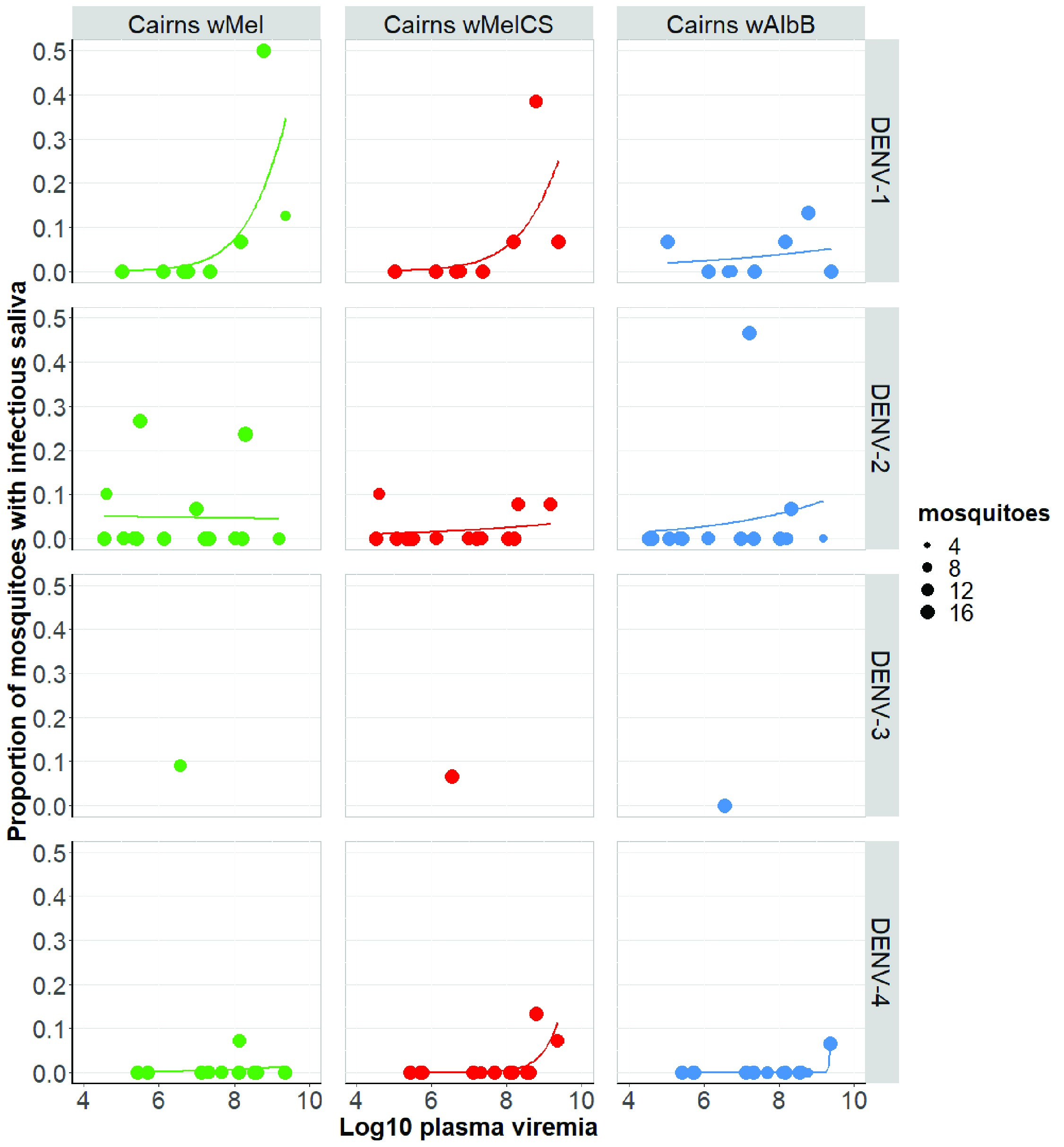

Supplement: S2 Fig — Each dot represented the proportion of each cohort that is infected, plotted as a function of log10 plasma viremia (RNA copies per milliliter), with the size of the dot indicative of the number of mosquitoes tested in each cohort, up to a maximum of 15. Data are stratified by the Wolbachia infection status, and the infecting serotype in the patient blood meal. (TIF) [file ppat.1008433.s006.tif]

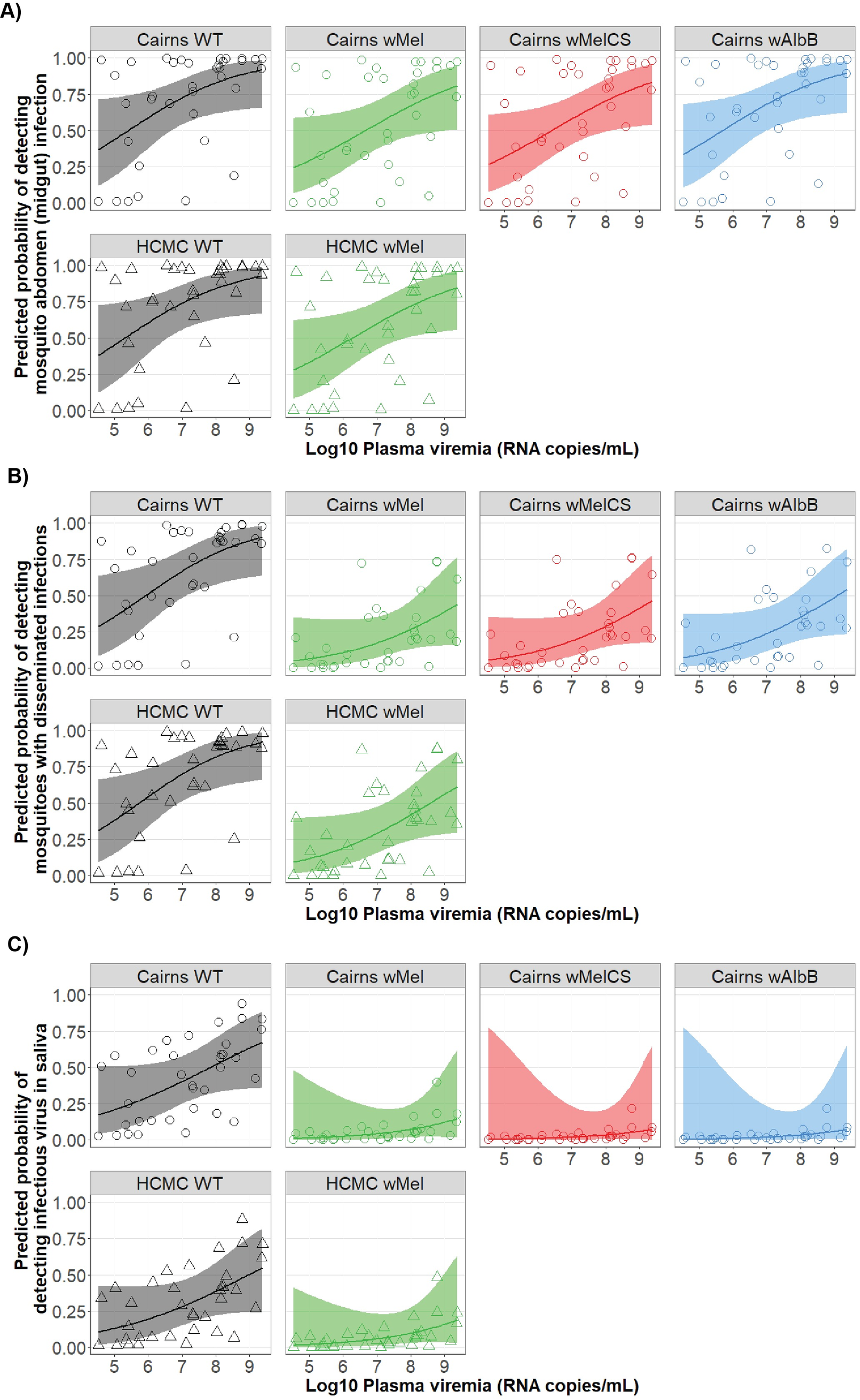

Supplement: S3 Fig — Predicted concentrations of virus leading to mosquitoes with DENV-positive (A) abdomens (representing midgut infections), (B) head/thorax (disseminated infections), and (C) infectious saliva (as measured in saliva-inoculated mosquitoes). Each point represents the predicted proportion of all mosquitoes to have DENV in the respective tissue tested, 14 days after a blood meal on a viremic blood from a dengue patient. The corresponding smoothing curves and shading (representing 95% CIs) illustrate the predicted probability based on marginal logistic regression. The point at which the smoothing curves cross the 50% on the y-axis represents the predicted concentration of virus required to infect 50% of mosquitoes (50% Mosquito Infection Dose; MID50). (TIF) [file ppat.1008433.s007.tif]
